# Editorial: Comparative Oncology–Advances in Veterinary Molecular Oncology

**DOI:** 10.3389/fvets.2021.812856

**Published:** 2022-01-12

**Authors:** Kristina Meichner, Angela L. McCleary-Wheeler, Hiroyuki Mochizuki, Tracy Stokol

**Affiliations:** ^1^Department of Pathology, College of Veterinary Medicine, University of Georgia, Athens, GA, United States; ^2^Department of Veterinary Medicine and Surgery, College of Veterinary Medicine, University of Missouri, Columbia, MO, United States; ^3^Department of Population Health and Pathobiology, College of Veterinary Medicine, North Carolina State University, Raleigh, NC, United States; ^4^Department of Population Medicine and Diagnostic Science, College of Veterinary Medicine, Cornell University, Ithaca, NY, United States

**Keywords:** cancer, genetics, pathogenesis, prognosis, targeted therapeutics

Molecular diagnostic assays are routinely performed in human oncology for tumor classification, prognostication, identification of therapeutic targets, and patient-specific therapy (1–3). In veterinary oncology, these molecular diagnostics have been limited to immunohistochemical (IHC) staining, flow cytometry (FC), and several PCR-based tests for detection of specific gene mutations (4), as seen in the report of this Research Topic. However, with high-throughput methodologies such as next-generation sequencing (NGS), the knowledge for molecular mechanisms in canine and feline cancers have been rapidly expanding in the past decade, discovering many potential biomarkers that can be translated into clinical applications.

Osteosarcoma is an aggressive bone tumor frequently affecting large-breed dogs. Luu et al. provide a comprehensive review on canine osteosarcoma focusing on potential tissue- and liquid biopsy (blood)-based biomarkers to aid in diagnosis, prognosis, and prediction and monitoring of treatment responses. Proteins that promote osteosarcoma growth and survival, e.g., epidermal growth factor receptor, human epidermal growth factor receptor-2 (HER2), and platelet-derived growth factor, are promising targets for treatment with tyrosine kinase receptor inhibitors and immunotherapy (e.g., Listeria vaccine targeting HER2) (5). However, based on this osteosarcoma review, currently there is no reliable diagnostic, prognostic, or predictive biomarker for canine osteosarcoma. Future studies should focus on technologies that are applicable in a clinical setting and move away from tissue- to blood-based biomarkers due to easier accessibility.

Flow cytometry is now routinely used to diagnose and phenotype canine lymphoma, being readily available to clinicians and less invasive than tissue sampling for histopathology. Rütgen et al. measured the cell proliferation marker Ki-67 by FC in neoplastic and non-neoplastic lymphocytes in dogs. Although counting mitotic figures in histopathologic sections is the “gold standard” for determining proliferation and tumor grade (6), Ki-67 is a more sensitive marker of cell proliferation, as it includes cells in all growth stages of the cell cycle (G1, S, G2, and M). Rütgen's group provided reference data for Ki-67 expression in non-neoplastic lymphocytes, which was significantly lower compared to neoplastic lymphocytes. Preliminary data showed that different lymphoma subtypes had varying degrees of Ki-67 expression. Future studies should determine whether FC-based Ki-67 expression could represent a low-invasive diagnostic tool to determine tumor grade and refine prediction of biologic behavior and prognosis in dogs with lymphoma.

The study by Heyward et al. had a lofty goal of finding an antibody that could be used to quantify circulating tumor cells (CTCs) in the blood of cats with mammary and oral squamous cell carcinomas (SCCs). Epithelial cellular adhesion molecule (EpCAM) expression was evaluated, because this transmembrane protein is used as a target to quantify epithelial CTCs in humans (7). However, the polyclonal anti-human EpCAM antibody that detected EpCAM in feline tumor cell lines with FC and in intestinal and mammary epithelia with IHC staining, failed to bind a high EpCAM-expressing oral SCC in static adhesion assays, indicating a need for higher quality reagents to detect and quantify CTCs in animals.

Feline mammary carcinoma (FMC) is one of the most common malignancies in cats. The clinicopathological and genetic similarities of FMC and human breast cancer (BC) support using the cat as a comparative model to discover novel biomarkers and therapeutic targets for human BC. The study by Gameiro et al. examined serum and tissue expression levels of leptin, an obesity-associated hormone, and its receptor (ObR). Similar to women with BC, serum ObR levels were increased in cats with FMC compared to healthy controls and were positively correlated with inflammatory cytokine levels. Leptin was also overexpressed in triple-negative FMC tissue, in concordance with human triple-negative BC. These results suggest that obesity-associated inflammation and altered leptin/ObR axis may contribute to FMC tumorigenesis. The leptin/ObR axis could be used as a diagnostic and prognostic biomarker and potential therapeutic target for FMC.

A case report of an unusual benign tumor of brown adipose tissue (BAT), a hibernoma, in a nipple was documented by Amorim et al.. This report with accompanying literature review highlights the importance of IHC for confirmatory diagnosis of this rare tumor. The distribution of hibernomas is not well-characterized, but it is typically found periorbital tissue, although this is not a location associated with BAT deposition. Because the histologic features of hibernomas–cells containing variably vacuolated abundant pale cytoplasm–can be mistaken for other adipose tumors, such as liposarcomas, positive IHC staining for uncoupling protein-1 is required for correct diagnosis.

The papers in this Research Topic illustrate the continued applicability of immunologic-based testing to characterize tumors in animals. However, the topic highlights the need for continued research into identification of useful cancer biomarkers and development of new reagents for tumor marker detection in domestic and exotic animals. Newer techniques that are being applied in humans, such as mutation detection, RNA sequencing, metabolomics, and proteomics are increasingly being used for these purposes in veterinary medicine. Specifically, NGS-based assays to detect circulating cancer cells or DNA fragments from cancer cells in fluid such as blood, called liquid biopsy, are still under investigation but showing early promise in human medicine (8). Liquid biopsy is non-invasive, allows serial sampling, and can be used not only for cancer diagnosis but also for disease monitoring and identification of prognostic and therapeutic markers (4, 9). Recent studies applied NGS and proteomic analysis to minimally invasive matrices such plasma and saliva to identify potential prognostic biomarkers and potential biomarkers to monitor therapeutic response in dogs with oral malignant melanoma (10, 11). Further studies are needed to validate putative diagnostic and prognostic molecular markers proposed so far and to answer questions of how molecular functions relate to oncogenic transformation, diagnosis, staging, treatment, and prognostication of tumors in veterinary medicine. Furthermore, bringing these high-throughput assays into clinical use with a standardized, quality-assured methodology will move us toward precision medicine.

## Author Contributions

KM, AM-W, HM, and TS wrote the manuscript. TS edited the manuscript. All authors contributed to the article and approved the submitted version.

## Conflict of Interest

The authors declare that the research was conducted in the absence of any commercial or financial relationships that could be construed as a potential conflict of interest.

## Publisher's Note

All claims expressed in this article are solely those of the authors and do not necessarily represent those of their affiliated organizations, or those of the publisher, the editors and the reviewers. Any product that may be evaluated in this article, or claim that may be made by its manufacturer, is not guaranteed or endorsed by the publisher.
